# Unique insertion/deletion polymorphisms within histidine-rich region of histidine-rich glycoprotein in Thoroughbred horses

**DOI:** 10.1038/s41598-023-27374-0

**Published:** 2023-01-06

**Authors:** Ryo Muko, Tomoya Sunouchi, Shuntaro Urayama, Yuko Toishi, Kanichi Kusano, Hiroaki Sato, Masanori Muranaka, Taekyun Shin, Masa-aki Oikawa, Yoshinobu Ojima, Mohammad Ali, Yoshihiro Nomura, Hiroshi Matsuda, Akane Tanaka

**Affiliations:** 1 Institute of Global Innovation Research, Tokyo University of Agriculture and Technology, Tokyo, Japan; 2 Laboratory of Comparative Animal Medicine, Division of Animal Life Science, Faculty of Agriculture, Tokyo University of Agriculture and Technology, 3-5-8 Saiwai-cho, Fuchu, Tokyo, 183-8509 Japan; 3 Race Horse Clinic, Ritto Training Center, Japan Racing Association, Shiga, Japan; 4 Shadai Stallion Station, Shadai Corporation, Hokkaido, Japan; 5 Race Integrity Section, Stewards Department, Japan Racing Association, Tokyo, Japan; 6 Department of Veterinary Anatomy, College of Veterinary Medicine and Veterinary Medical Research Institute, Jeju National University, Jeju, South Korea; 7 Diagnostic Laboratory, Equine Veterinary Medical Center, Education City, Doha, Qatar; 8 Scleroprotein and Leather Research Institute, Faculty of Agriculture, Tokyo University of Agriculture and Technology, Tokyo, Japan; 9 Cooperative Major in Advanced Health Science, Graduate School of Bio-Applications and System Engineering, Tokyo University of Agriculture and Technology, Tokyo, Japan

**Keywords:** Molecular biology, Zoology, Medical research

## Abstract

Histidine-rich glycoprotein (HRG) is abundant plasma protein with various effects on angiogenesis, coagulation, and immune responses. Previously, we identified the base and amino acid sequences of equine HRG (eHRG) and revealed that eHRG regulates neutrophil functions. In this study, we first conducted a large-scale gene analysis with DNA samples extracted from 1700 Thoroughbred horses and identified unique insertion/deletion polymorphisms in the histidine-rich region (HRR) of *eHRG*. Here we report two types of polymorphisms (deletion type 1 [D1] and deletion type 2 [D2]) containing either a 45 bp or 90 bp deletion in the HRR of *eHRG*, and five genotypes of *eHRG* (insertion/insertion [II], ID1, ID2, D1D1, and D1D2) in Thoroughbred horses. Allele frequency of I, D1, and D2, was 0.483, 0.480, and 0.037 and the incidence of each genotype was II: 23.4%, ID1: 46.2%, ID2: 3.6%, D1D1: 23.1%, and D1D2: 3.7%, respectively. The molecular weights of each plasma eHRG protein collected from horses with each genotype was detected as bands of different molecular size, which corresponded to the estimated amino acid sequence. The nickel-binding affinity of the D1 or D2 deletion eHRG was reduced, indicating a loss of function at the site. eHRG proteins show a variety of biological and immunological activities in vivo, and HRR is its active center, suggesting that genetic polymorphisms in *eHRG* may be involved in the performance in athletic ability, productivity, and susceptibility to infectious diseases in Thoroughbred horses.

## Introduction

Much is known regarding humans have made use of many animals throughout the history of the evolution. It is now believed that horses were domesticated in the steppe regions of Western Eurasia, especially in the lower Volga Don, between the late 4th and early third centuries BC^1^. In the First World War, more than 1.2 million horses and mules served in the British army and 484,000 died on the battlefield; surviving animals were additionally sold overseas for labor or meat^2^. According to their contributions to our history, we should engage in the proper maintenance and management of horses, because they are important companions that help, entertain, and heal people in animal-assisted therapy or horse business. Infection, trauma, anaphylaxis, ischemia, and hemorrhage occasionally cause life-threatening conditions such as systemic inflammatory response syndrome (SIRS), sepsis, or endotoxemia^3^. Despite the high fatality rate following an extensive economic loss, there is currently no curative treatment for these disorders. Moreover, histidine-rich glycoprotein (HRG) has been reported to be a promising biomarker for human SIRS and sepsis^4,5^.

Human HRG is a 75 kDa plasma glycoprotein synthesized in liver parenchymal cells and is present in human plasma at concentrations of approximately 100–150 μg/mL^6–8^. HRG is a multidomain polypeptide consisting of two cystatin-like regions at the N-terminal, a histidine-rich region (HRR) flanked by two proline-rich regions (PRR1 and PRR2), and a C-terminal domain^6^. Various molecules interacting with HRG have been defined, such as heparin, phospholipids, plasminogen, fibrinogen, immunoglobulin G, C1q, heme, and zinc ions^9–13^. Based on these interactions, HRG is assumed to have biological functions in the immune response, coagulation, and angiogenesis^6,14^. HRG is also involved in the pathogenesis of SIRS and sepsis, and in human patients, plasma HRG levels in non-survivors have been reported to be lower than those in survivors^4,5^. Moreover, therapeutic possibility of HRG has been demonstrated by administrating purified human HRG to a mouse model of sepsis^15^. Therefore, HRG may serve as a prognostic biomarker as well as one of novel pharmaceutical reagents for SIRS and sepsis^4,5,15,16^.

To date, several polymorphisms in human HRG have been reported. Patients with Tokushima-1 mutation (NP_000403.1: p.Gly103Glu, rs121918122) had low plasma HRG levels (21%) and suffered from right transverse sinus thrombosis^7,17^. Patients with Tokushima-2 mutation (NP_000403.1: p.Cys241Arg, rs2276804) had plasma HRG deficiency (50%) and suffered from a Dural arteriovenous fistula^7^. The amino acid substitution of HRG (NP_000403.1: p.Pro204Ser, rs9898) is involved in male and female infertility^18,19^. The rs9898 missense mutation results in an additional glycosylation site at position 202 of the HRG. This single nucleotide polymorphism (SNP) is relatively common, with a frequency of 0.49 for the Ser allele and 0.51 for the Pro allele^20^. Male homozygous carriers of the SNP showed lower total sperm counts, sperm concentrations, motility scores, yields after preparation, and pregnancy rates following in vitro fertilization^18^. Also, *HRG* rs9898 exhibited a decrease in activated partial thromboplastin time (aPTT), resulting in susceptibility to thrombotic diseases^21,22^. Based on these reports, genetic mutation of *HRG* may be involved in its protein activity and risks of certain diseases. However, gene polymorphisms of *equine HRG* (*eHRG*) and the functions of each eHRG protein have not been explored yet.

We started to investigate eHRG as possible indicators of SIRS and endotoxemia, and first reported gene sequences and detected proteins from plasma of horses. We have also revealed that eHRG showed various effects on neutrophil functions in horses^23,24^. Within the present study, we conducted a large-scale analysis with 1700 Thoroughbred horses and found two deletions at position 1120–1164 (D1) and 1075–1164 (D2) which is within HRR and five genotypes of *eHRG* that were unique to horses. In addition, we revealed that eHRG proteins from horses of each genotype had different molecular weights. Our findings may contribute to the further elucidation of new biological significance of HRG from the perspective of comparative medicine.

## Results

### Polymorphisms of *eHRG*

**Figure 1:**
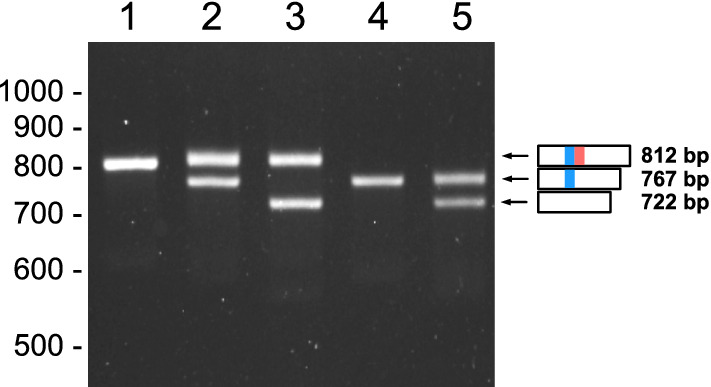
Agarose gel electrophoresis patterns of e*HRG* polymorphisms. The HRR of e*HRG* was amplified by PCR using genomic DNA extracted from the leukocytes. The PCR products were separated on 2% agarose gel and stained with ethidium bromide. Five amplifying band patterns consisting of three bands of different lengths were detected. Lanes 1 and 4: homozygous genotypes; lanes 2, 3, and 5: heterozygous genotypes. The photo represents a typical image obtained from the PCR experiments of 1700 Thoroughbred horses.

We have already reported that mRNA production of *eHRG* was detected only in the liver of horses^23^. Using liver cDNA from Thoroughbred horses, the nucleic acid and amino acid sequences of *eHRG* were identified^23^. In the present study, by using this information, a large-scale analysis of Thoroughbred *eHRG* genotypes was carried out during May 2018–July 2022 and partial deletions in the HRR of *eHRG* gene in some horses were found. To examine these deletions, a polymerase chain reaction (PCR) method amplifying the part of the *eHRG* sequence containing HRR using genomic DNA from leukocytes was established. Using this method, the genotypes of 1700 horses were analyzed, and 5 amplifying bands with different base pairs (bp) were identified (Fig. 1). A band containing all the sequences was detected at position 812 bp (I). However, bands at 767 and 722 bp, which differed from those, were also detected. During analysis, we found combinations of five different genotypes: 812 bp alone (lane 1), 812 bp and 767 bp (lane 2), 812 bp and 722 bp (lane 3), 767 bp alone (lane 4), and 767 bp and 722 bp (lane 5) (Fig. 1). All 1700 horses analyzed in this study had one of these five genotypes. However, no horse with a single band of 722 bp was detected.

### Sequence identification of *eHRG* polymorphism

**Figure 2:**
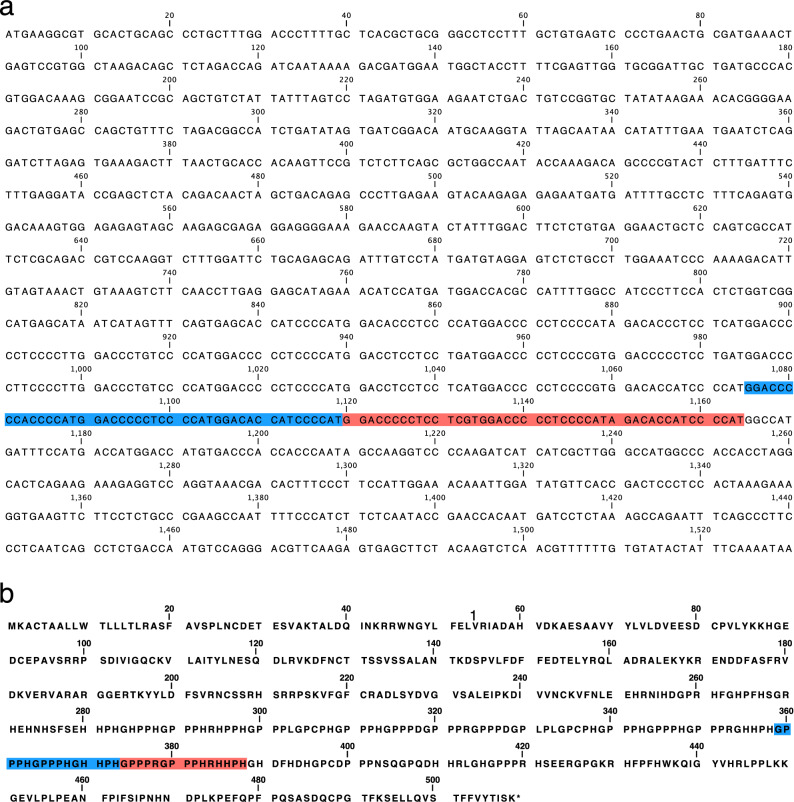
Nucleotide and deduced amino acid sequences of e*HRG* gene. (**a**) Nucleotide sequence of cloned e*HRG* gene. 45 bp deletion: red highlighting area; 90 bp deletion: blue and red highlighting area. (**b**) Deduced amino acid sequence of e*HRG*. 15 amino acid deletion: red highlighting area; 30 amino acid deletion: blue and red highlighting area.

**Figure 3:**
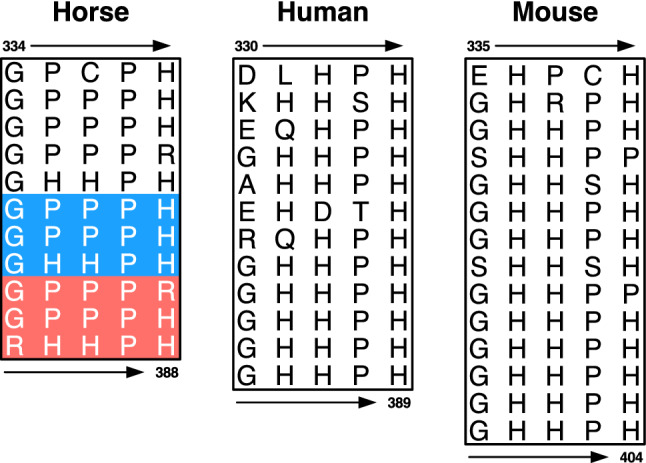
Amino acid sequence of HRR in horse, human, and mouse *HRG* gene. Different types of tandem repeats in the HRR of *HRG* gene are found. Amino acid sequences vary among 3 species. Alphabets in the figure indicate the symbols for the various amino acids. 45 bp deletion: red highlighting area; 90 bp deletion: blue and red highlighting area. The GenBank accession numbers are as follows: human, NP_000403; mouse, NP_444406.

To determine the sequence of each band detected in Fig. 1, the band amplified by PCR was separated by 2% agarose gel electrophoresis and each band was extracted. The extracted samples were sequenced, and two types of deletions, a 45 bp deletion (D1; LC723849.1:c.1120_1164del) and a 90 bp deletion (D2; LC723849.1:c.1175_1164del) were identified in the HRR of *eHRG*. The nucleic acid and amino acid sequences of each deletion are shown in Fig. 2a, b, respectively. The full-length HRR of *eHRG* (LC723849.1) is composed of 11 tandem repeats with 15 bases that encode 5 amino acids, as shown in Fig. 3. D1 lacks three repeats of 45 bps (red area), and D2 lacks six repeats of 90 bps (blue and red areas). According to the results, the five genotypes of genetic polymorphisms shown in Fig. 1 were named as II, ID1, ID2, D1D1, and D1D2.

The amino acid sequence of the human and mouse HRR domains is composed of repeats of the consensus sequence Gly-His-His-Pro-His (GHHPH) with 12 (60 aa) repeats in humans and 14 (70 aa) repeats in mice (Fig. 3). In contrast, the consensus sequence in the HRR of horse HRG revealed in this experiment is Gly-Pro-Pro-Pro-His (GPPPH), which is composed of 11 (55 aa) consensus sequence repeats (Fig. 3). The proportion of histidine residues in the HRG protein was examined and found to be 9.2% for horses, 12.6% for humans, and 11.0% for mice, respectively^7^. The proportion of histidine residues was lower in horses with insertion type eHRG than in humans and mice, whereas the proportion of proline residues was 16.3%, 12.4%, and 9.9% in horses, humans, and mice, respectively, indicating a tendency for more proline residues in horses than in humans and mice^7^.

### Polymorphism frequency of *eHRG*

We analyzed genetic polymorphisms using genomic DNA purified from the blood of 1700 Thoroughbred horses, and the number and frequency of each genetic polymorphisms are shown in Table 1. The most common genotype, ID1 occupied approximately half of samples (46.2%), and the second most common were II and D1D1 (23.4% and 23.1%, respectively). The frequencies of ID2 and D1D2 were lower (3.6% and 3.7%, respectively). As these genotypes are present in more than 1%, these deletions can be defined as genetic polymorphisms. No gender differences were found in the occurrence of each genotype, with the highest proportion of ID1 individuals in each case. In contrast, no horse with a homozygous 90 bp deletion (D2D2) in *eHRG* was found. The expected gene frequency of I, D1, and D2, was 0.483, 0.480, and 0.037, respectively. Predicted values for each genotype were calculated from the gene frequency data. As shown in Table 2, the predicted values correlated with the actual values. According to the predicted values, more than two D2 homozygotes should have been found in 1700 horses, but these were not found in this study.

**Table 1:** Incidence of insertion/deletion polymorphism of *eHRG* gene in Thoroughbred horses.

Genotype	Gender						Total (n = 1700)	
	Male (n = 936)		Female (n = 686)		Gelding (n = 78)			
	n	%	n	%	n	%	n	%
II	211	22.5	163	23.8	24	30.8	398	23.4
ID1	438	46.8	314	45.8	33	42.3	785	46.2
ID2	34	3.6	25	3.6	3	3.8	62	3.6
D1D1	214	22.9	161	23.5	17	21.8	392	23.1
D1D2	39	4.2	23	3.4	1	1.3	63	3.7
D2D2	0	0	0	0	0	0	0	0

II, Insertion/insertion; ID1, Insertion/45 bp deletion; ID2, Insertion/90 bp deletion; D1/D1, 45 bp deletion/45 bp deletion; D1D2, 45 bp deletion/90 bp deletion; D2D2, 90 bp deletion/90 bp deletion; N, Numbers of horses; %, Percentage.

**Table 2:** Actual and expected values of each *eHRG* genotype in Thoroughbred horses.

Genotype	Actual values		Expected values	
	n	%	n	%
II	398	23.4	397.0	23.4
ID1	785	46.2	788.6	46.4
ID2	62	3.6	60.4	3.6
D1D1	392	23.1	391.7	23.0
D1D2	63	3.7	60.0	3.5
D2D2	0	0	2.3	0.1

II, Insertion/insertion; ID1, Insertion/45 bp deletion; ID2, Insertion/90 bp deletion; D1/D1, 45 bp deletion/45 bp deletion; D1D2, 45 bp deletion/90 bp deletion; D2D2, 90 bp deletion/90 bp deletion; N, Numbers of horses; %, Percentage.

### eHRG detection in plasma

**Figure 4:**
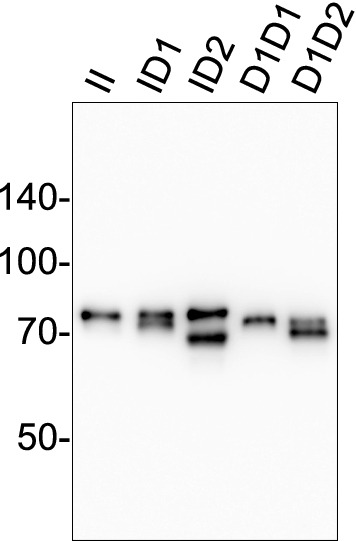
Molecular weight and migration pattern of plasma eHRG with 5 genotypes. Plasma of each genotype was selected from the genotyping results and eHRG protein was detected by western blotting. Shown are presentative photo of five independent experiments. The numbers on the left are molecular weight markers. II: Insertion/insertion, ID1: insertion/45 bp deletion, ID2: insertion/90 bp deletion, D1/D1: 45 bp deletion/45 bp deletion, D1D2: 45 bp deletion/90 bp deletion.

To investigate whether each genotype detected by PCR reflected protein phenotypes in plasma, eHRG in plasma was detected by western blotting. Plasma HRG proteins from each genotype had a different band pattern; homozygous genotypes (II and D1D1) showed a single band and heterozygous genotypes (ID1, ID2, and D1D2) showed doublet bands (Fig. 4). The estimated molecular weight of insertion-type (I) was 77.6 ± 1.6 kDa, D1 was 73.1 ± 1.5 kDa, and D2 was 68.1 ± 2.2 kDa, respectively. The estimated molecular weights using the amino acid sequences of insertion-type (I) and deletion-type (D1 or D2) eHRGs were I-type: 56.7 kDa, D1:55.1 kDa, and D2:53.5 kDa, respectively. The molecular weight of each molecule detected by western blotting was higher than the molecular weights calculated from the amino acid sequences.

### Binding affinity of eHRG with Ni-sepharose

Because histidine has a high affinity for divalent cations, HRG can be purified from plasma using Ni-sepharose^25,26^. Each deletion genotype of *eHRG*, D1, and D2, contains a lesser amount of histidine residues than full-length eHRG (Insertion: 9.2% [47 aa], Deletion 1: 8.7% [43 aa], Deletion 2: 7.9% [38 aa]). Thus, we extracted eHRG from plasma of horses with deletion genotypes (ID1, ID2, D1D1, and D1D2) and compared with that from the II genotype. There was no significant difference in the total amount of plasma protein between the groups (Fig. S3a). The Ni-binding protein yield from the plasma of the ID1 or ID2 genotypes was lower than that of II (Fig. S3b), but it was not statistically significant. However, the yield of Ni-binding protein was significantly lower in D1D1 and D1D2 genotype, comparing to that in II type (Fig. S3b).

### Enzyme-linked immunosorbent assay (ELISA) for eHRG in plasma

**Figure 5:**
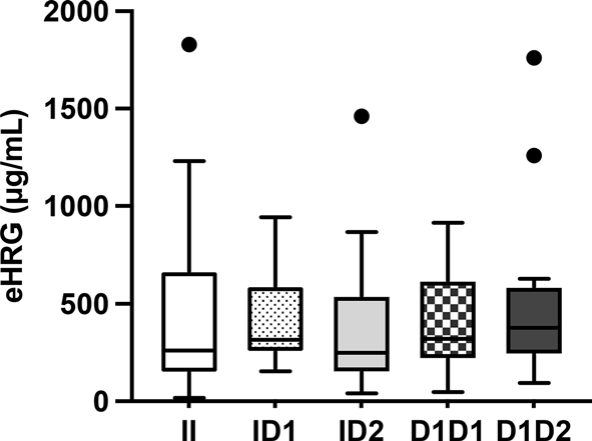
Plasma levels of eHRG protein. Plasma levels of eHRG were measured by an ELISA. Obtained data were shown using a box plot that is composed of the median (solid line in each column), upper hinge, lower hinge, whiskers representing upper adjacent value or lower adjacent value. Black dots above columns indicate far out values. N = 20 in each group. II: Insertion/insertion, ID1: insertion/45 bp deletion, ID2: insertion/ 90 bp deletion, D1/D1: 45 bp deletion/45 bp deletion, D1D2: 45 bp deletion/90 bp deletion.

Finally, a competitive ELISA was performed with the collected plasma to determine if there were differences in the concentrations of HRG in the blood of horses with each genotype. An ELISA was performed in duplicates on 20 horses with each genotype to obtain data using a rabbit polyclonal anti-eHRG antibody generated as described in method section. Measurement data showed the median [interquartile range] as follows: II:261 [148–668], ID1:315 [255–591], ID2:249 [150–541], D1D1:320 [218–619], and D1D2:378 [240–587] μg/mL) (Fig. 5). There were no significant differences in measurements between the groups.

## Discussion

In this study, two deletions (LC723849.1:c.1120_1164del and LC723849.1:c.1175_1164del) in the HRR of *eHRG* were identified in Thoroughbred horses. These deletions result in five genotypes, named within the study: II, ID1, ID2, D1D1, and D1D2. The HRG gene has been analyzed in humans, mice, rabbits, and several other animals^6,7^. Single nucleotide mutations and SNPs were reported to be involved in the downregulation of plasma HRG levels, infertility, and thrombus formation in humans^7,17–19,22^. However, there is no report on animals that have such deletion polymorphisms in HRR like horses. Since the frequency of the D2 deletion type is 0.037, D2 homozygous individuals should be found in 1–2 out of every 1000 horses. Despite conducting a multi-sample analysis with 1700 Thoroughbred horses, we could not find any horses with a homozygous genotype of D2. Therefore, homozygous D2 carriers might have some abnormalities in embryogenesis, development, or low potential of racing horses, such as having poor growth or being susceptible to infectious diseases. Since the gene frequency of D2 is lower than that of the others, more sample analysis may be necessary to reach this conclusion. eHRG concentration in plasma was evaluated by competitive ELISA, and there was no significant difference among horses with each genotype. The plasma level of eHRG is approximately 300 μg/mL, which is 2–3 times higher than that in humans (100–150 μg/mL) and mice (75–150 μg/mL)^6–8,27^. Concentration differences among different animal species is one of the tasks for future study.

HRG is the histidine residue-rich protein, with human HRG containing 66 histidine residues out of 525, approximately 13% of all. They are concentrated in the HRR of the six domains, with 53% of the HRR of human HRG being histidine residues. Because histidine has a high affinity for divalent cations, HRG can be purified from plasma using Ni-sepharose^25,26^. Each deletion genotype of *eHRG*, D1 and D2, contains less histidine residues than full-length *eHRG* (Insertion: 9.2% [47 aa], Deletion 1: 8.7% [43 aa], Deletion 2: 7.9% [38 aa]). Thus, plasma from horses with deletion genotypes must contain a lower amount of Ni-sepharose binding protein than plasma from the II genotype. In fact, we confirmed that the amount of Ni-sepharose binding protein in the plasma was different among *eHRG* genotypes (Fig. S3b), indicating that the genotypes may affect eHRG binding with divalent cations. Interestingly, the eHRG yields of ID1 and ID2, and those of D1D1 and D1D2, were not different. The higher amount of histidine lost in the D2-deficient form suggests that the histidine lost in the D1-deficient form binds primarily to nickel, while the region lost only in the D2-deficient form may be structurally less likely to bind nickel. Since the interaction between HRG and its ligands is mediated by binding of Zn^2+^ to the histidine residue of the HRR modification^6,12,28,29^, mutations in HRR may alter HRG function. Additionally, protonation of histidine residues under acidic conditions prevents nitrogen from participating as a ligand donor for Zn^2+^, indicating that HRG functions are also affected by environmental pH^12,30,31^. The predicted isoelectric points (pIs) of eHRG were 7.53 (insertion), 7.15 (deletion 1), and 7.10 (deletion 2); the isoelectric point of the deletion-type eHRGs is lower than that of the insertion-type eHRG. The difference in isoelectric points demonstrates that insertion-type eHRG tends to be protonated compared with the deletion-type eHRGs under acidic condition. On the other hand, the pH range of Zn^2+^ binding with the deletion-type eHRGs would be broader than that of insertion-type eHRG. Furthermore, Zn^2+^ and pH are involved in the disassembly of HRG; plasmin cleavage of HRG is inhibited by acidic pH, and the trypsin cleavage pattern of HRG is altered by the presence of Zn^2+^^30,32^. The formation of the immuno-thrombus at the inflammation site leads to platelet activation, resulting in the increase in Zn^2+^ concentration at the affected sites^12^. Since the pH of wound sites becomes acidic, the interaction between HRG and ligands and cleavage of HRG must modify the antimicrobial and angiogenic properties of HRG^12,33–38^. Deletion-type eHRGs may cause a delay in protonation and limitation in functions of proteins compared with that of insertion-type. Insertion-type eHRGs and deletion-type eHRGs may differ in their adaptation to and function in inflammatory conditions. These differences may explain why several genotypes of eHRG are conserved in horses.

Since the deletion sites of HRR in *eHRG* do not contain N-linked glycosylation sites, insertion-type (I) and deletion-type (D1 and D2) eHRGs are expected to undergo similar glycosylation if there are no conformational changes due to gene deletion. The molecular weight of each protein detected by western blotting was higher than each molecular weight estimated from the amino acid sequence, possibly because of protein glycosylation. However, the difference in molecular weights between the assumed proteins and the final products was found to vary by genotype. These observations suggest that genotypic variation affects the three-dimensional structure of HRG, which may be responsible for the differences in glycosylation and molecular weight of each eHRG.

In the current study, we found five different genotypes in the e*HRG* of Thoroughbred horses, showing that the genotype correlates with the molecular weight of the eHRG protein. As we have already reported that eHRG functions as a dual regulator of neutrophil activity in horses, the genotype of *eHRG* may affect certain characteristics of horses, including disease susceptibility, athletic performance, and reproduction. Follow-up studies on genotyping of *eHRG* as well as functional and conformational analysis of the deletion-type *eHRGs* should be provided. Moreover, a large-scale analysis of other horse breeds must be conducted; however, our findings may provide a new perspective on the relationship between HRG and horse characteristics.

## Methods

### Ethics statement

All experiments with Thoroughbred horses were performed according to the standards specified in the guidelines provided by the Animal Care and Use Committee of the Tokyo University of Agriculture and Technology, as well as those in both the Japan Racing Association (JRA, Shiga, Japan) and Shadai Stallion Station (SSS, Hokkaido, Japan) guidelines for sample collection from horses. Blood collection from horses was carried out in accordance with guidelines and regulations of Tokyo University of Agriculture and Technology, JRA, and SSS. All methods are reported in accordance with ARRIVE guidelines. The entire study was approved by the Animal Care and Use Committee of the Tokyo University of Agriculture and Technology (approval nos. 30–104, R02-46, R03-50, and R04-85).

### Polymorphism analysis of eHRG

Blood samples were collected from the jugular vein of 1700 clinically healthy Thoroughbred horses (936 males, 78 geldings, and 686 females, aged 2–28 years, mean age ± SD = 4.0 ± 2.4) using heparinized tubes during annual laboratory tests for monitoring infectious diseases at the Ritto Training Center of the JRA and SSS from May 2018 to July 2022. The samples were stored at 4 °C, and leukocytes were collected within a couple of days.

*eHRG* polymorphisms were analyzed using genomic DNA purified from horse leukocytes. Briefly, blood was centrifuged at 1500 rpm for 15 min at 4 °C, and the plasma and blood cells were separated. Leukocytes isolated from buffy coats were used for DNA analysis, and plasma was stored at − 80 °C until protein analysis. DNA was extracted from leukocytes in the buffy coat using Maxwell® 16 Blood DNA Purification or Maxwell^®^ RSC Tissue DNA Kit (Promega, Madison, WI, UAS), according to the manufacturer’s protocol. Extracted DNA samples were used for polymerase chain reaction (PCR) with GoTaq® Master Mix (Promega) and a pair of primers (Forward: 5′-ACTCTGGTCGGCATGAGCATA-3′, Revers: 5′-TTTGTGTTTATTACTGGTCACATT-3′). The PCR products were separated using 2% agarose gel electrophoresis and visualized with ethidium bromide. Allele frequencies were estimated using the gene-counting method, and Hardy–Weinberg equilibrium was performed using allele frequencies.

### Sequencing of the deletion site within HRR of eHRG

Amplified PCR products were separated by 2% agarose gel electrophoresis and each migrated band was cut and dissolved in Buffer A (GL Sciences, Tokyo, Japan). The PCR product was extracted from the solution using Wizard® SV Gel and PCR Clean-Up System (Promega) and purified PCR products were inserted into a pGEM-T TA cloning vector according to the manufacturer's instructions, followed by PCR using the BigDye Terminator v3.1 Cycle Sequencing kit (Life Technologies, Grand Island, NY, USA) with the primer 5′-TAGAAGCTCACTCTTGAACGT-3′. The PCR products were analyzed using an ABI Avant-3100 sequencer (Life Technologies), according to the manufacturer's instructions. Protein pIs of wild-type and deletion type eHRG were determined with the Compute pI/Mw tool at ExPASy (https://web.expasy.org/compute_pi/) using each *eHRG* sequence.

### Detection of eHRG protein with different genotypes by western blotting

To study the effect of each genotype on protein production, eHRG in the plasma of horses with each genotype was detected using western blotting. Heparinized Thoroughbred bloods were centrifuged at 1500 rpm for 15 min at 4 °C and the collected plasma was analyzed by western blotting as described previously using murine anti-HPRG monoclonal antibody (Santa Cruz, Santa Cruz, CA, USA; G-10), secondary anti-mouse IgG antibody (Cell Signaling Technology, Beverly, MA, USA), and HRP-linked secondary antibody (Cell Signaling Technology). The primary antibody is a mouse monoclonal antibody, whose epitope is part of the human HRG sequence (25 amino acids in N-terminal domain 1, 96% homology to horse). Positive bands were visualized with an LAS-4000 (Fuji Film, Tokyo, Japan)^23^. The molecular weight of each band was calculated using ImageJ with reference to the technical brief supplied by Bio-Rad Laboratories Inc. (Bio-Rad Laboratories, Hercules, CA, USA).

### Preparation of rabbit polyclonal anti-eHRG antibody

An eHRG-specific antibody was produced in rabbits by immunization with a synthetic 22 amino-acid peptide (PRHSEERGPGKRH) from PRR2 of the eHRG sequence. Rabbit polyclonal anti-eHRG antibody was purified from the serum using antigen affinity purification. Immunoreactivity was confirmed by immunoblotting with purified eHRG^23^.

### Competitive enzyme-linked immunosorbent assay (ELISA)

Plasma eHRG concentrations were determined using an in-house competitive ELISA. Briefly, a 96-well polystyrene plate (Cat. no. 9018, Corning, NY, USA) was precoated with 100 μL of 0.5 μg/mL purified eHRG in phosphate buffered saline (PBS) and incubated overnight at 4 °C. After incubation, the plate was washed three times with wash buffer (0.05% Tween-20 in PBS). Nonspecific binding was blocked with 300 μL blocking buffer (1% bovine serum albumin in 0.05% Tween-20 in PBS) for 1 h at room temperature. The blocking buffer was discarded, and each well was washed three times with wash buffer. After washing, 100 μL of 20-fold diluted equine plasma from each genotype (n = 20) and 2 μg/mL polyclonal rabbit anti-eHRG antibody was added to each well and incubated for 1 h at room temperature. Purified eHRG (0–100 μg/mL) was used as the standard. The standards and samples were diluted with blocking buffer containing a complete protease inhibitor cocktail (Roche, Basel, Switzerland). Subsequently, the plate was washed with wash buffer, followed by the addition of 100 μL biotinylated anti-rabbit IgG (Cell Signaling Technology, MA, USA) in blocking buffer and incubation for 1 h at room temperature. After incubation, each well was washed thrice with wash buffer, and 100 μL piece streptavidin poly-HRP (Thermo Fisher Scientific) was added and incubated for 30 min at room temperature. Then, the plate was washed with wash buffer followed by the addition of 100 μL TMB Substrate OptEIA (BD, NJ, USA), and color development was stopped by the addition of 100 μL 0.5 N H_2_SO_4_. The absorbance was measured at 450 nm, and a standard curve was generated following the calculation of plasma eHRG concentrations.

### HRG purification from equine plasma

eHRG purification was performed using a previously described method, with slight modifications^15,23,24^. Briefly, the collected equine plasma was incubated with Ni Sepharose 6 Fast Flow (GE Healthcare, Piscataway, NJ, USA) in the column for 10 min at room temperature. After incubation, the mixture was washed twice, once with 50 mM Tris–HCl (pH 7.5) containing 0.5 M NaCl and 20 mM imidazole, and second with 50 mM Tris–HCl (pH 7.5) containing 0.5 M NaCl and 100 mM imidazole. Then the binding protein with sepharose was eluted with 50 mM Tris–HCl (pH 7.5) containing 0.5 M NaCl and 300 mM imidazole. The eluted solution was further purified using an Amicon Ultra 0.5 mL 30 kDa centrifugation filter (Millipore, Bedford, MA, USA). The protein yield in plasma and collected samples was measured using the Pierce™ BCA Protein Assay Kit (Thermo Fisher Scientific, MA, USA). Ni-binding protein was expressed as weight per mg of total plasma protein.

### Statistics

Dunnett’s multiple comparison was performed using Graph Pad Prism 9 Version 9.4.1 for the statistical analysis of the data, and *p* values < 0.05 were considered statistically significant.

## Supplementary Information

Supplementary Information.

## Data Availability

The mRNA sequences of insertion/deletion types of *eHRG* (I, D1, and D2) are available in DNA DataBank of Japan (DDBJ)/European Nucleotide Archive (ENA)/GenBank. Accession numbers of I, D1, and D2 are LC723849, LC726342, and LC726343, respectively (link to “http://getentry.ddbj.nig.ac.jp/top-e.html”). Human HRG SNP information of rs9898 was obtained from Ensembl version 108 (https://asia.ensembl.org/Homo_sapiens/Variation/Explore?r=3:186672338-186673338;v=rs9898;vdb=variation;vf=90106483). All data used or analyzed during this study are available from the corresponding author on reasonable request.
