# Supplementary material for: Unique insertion/deletion polymorphisms within histidine-rich region of histidine-rich glycoprotein in Thoroughbred horses
Source: Sci Rep. 2023 Jan 6;13:300. doi: 10.1038/s41598-023-27374-0 (PMC9822902; doi:10.1038/s41598-023-27374-0)
Supplement: Supplementary file 1 — Supplementary Information. [file 41598_2023_27374_MOESM1_ESM.docx]

**Unique insertion/deletion polymorphisms within histidine-rich region of histidine-rich glycoprotein in Thoroughbred horses**

Ryo Muko^1^, Tomoya Sunouchi^2^, Shuntaro Urayama^3^, Yuko Toishi^4^, Kanichi Kusano^3^, Hiroaki Sato^5^, Masanori Muranaka^3^, Taekyun Shin^6^, Masa-aki Oikawa^7^, Yoshinobu Ojima^1^, Mohammad Ali^7^, Yoshihiro Nomura^8^, Hiroshi Matsuda^2^ and Akane Tanaka^1, 2, 9 *^

^1^Institute of Global Innovation Research, Tokyo University of Agriculture and Technology, Tokyo, Japan

^2^ Laboratory of Comparative Animal Medicine, Division of Animal Life Science, Faculty of Agriculture, Tokyo University of Agriculture and Technology, Tokyo, Japan.

^3^ Race Horse Clinic, Ritto Training Center, Japan Racing Association, Shiga, Japan.

^4^ Shadai Stallion Station, Shadai Corporation, Hokkaido, Japan.

^5^ Race Integrity Section, Stewards Department, Japan Racing Association, Tokyo, Japan

^6^ Department of Veterinary Anatomy, College of Veterinary Medicine and Veterinary Medical Research Institute, Jeju National University, Jeju, South Korea.

^7^ Diagnostic Laboratory, Equine Veterinary Medical Center, Education City, Doha, Qatar.

^8^ Scleroprotein and Leather Research Institute, Faculty of Agriculture, Tokyo University of Agriculture and Technology, Tokyo, Japan.

^9^ Cooperative Major in Advanced Health Science, Graduate School of Bio-Applications and System Engineering, Tokyo University of Agriculture and Technology, Tokyo, Japan.

*Correspondence and requests for materials should be addressed to Akane Tanaka, Laboratory of Comparative Animal Medicine, Division of Animal Life Science, Faculty of Agriculture, Tokyo University of Agriculture and Technology, 3-5-8 Saiwai-cho, Fuchu, Tokyo 183-8509, Japan.

email address: akane@cc.tuat.ac.jp (A. Tanaka).


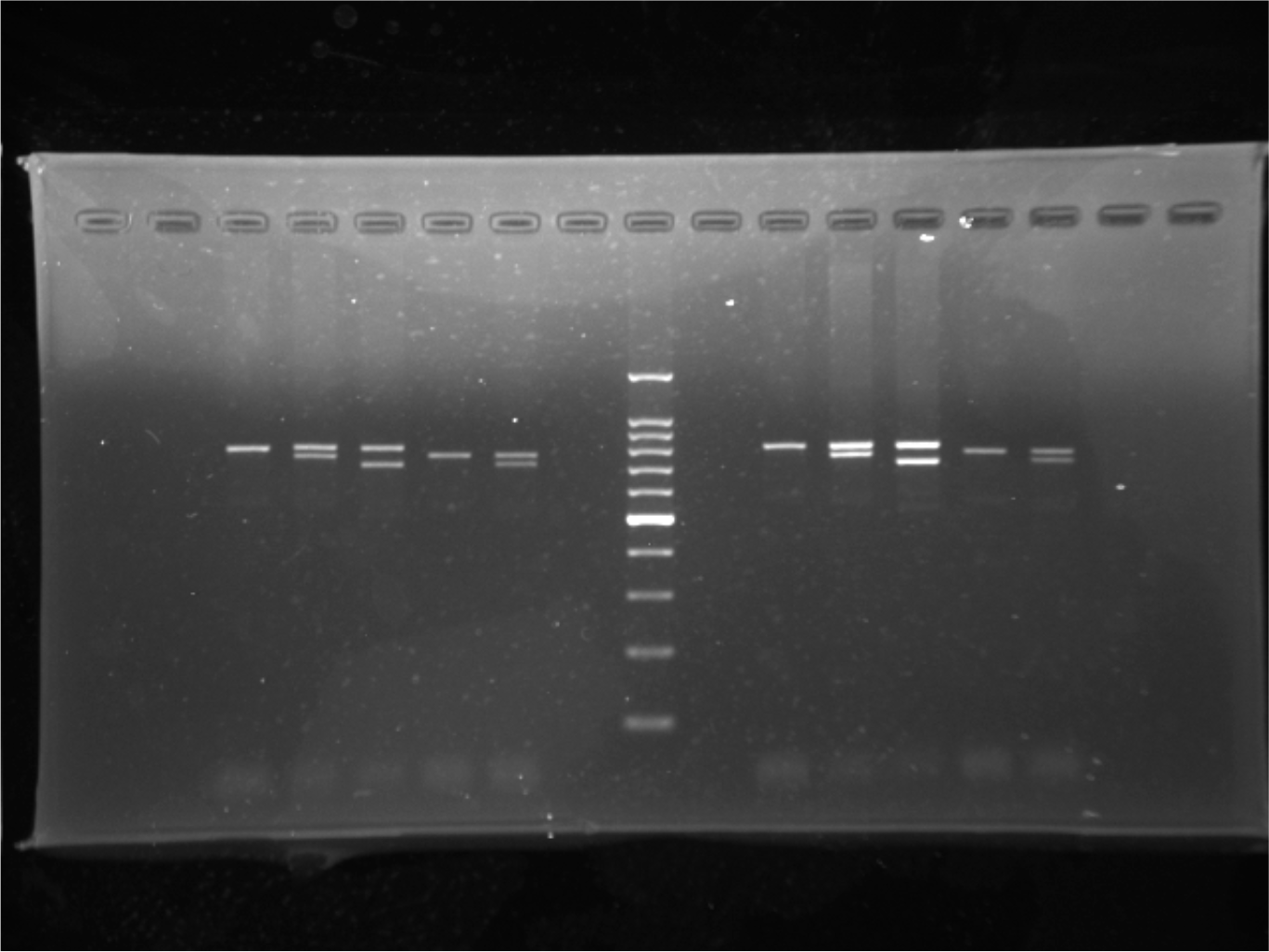


Figure S1

Full length image of agarose gel electrophoresis patterns of e*HRG* polymorphisms. The HRR of e*HRG* was amplified by PCR using genomic DNA extracted from the leukocytes. The PCR products were separated on 2% agarose gel and stained with ethidium bromide. Five amplifying band patterns consisting of three bands of different lengths were detected at lane 3 – 7 and 11 – 15. Center lane (lane 9) indicates ladder marker. Left (lane 3 – 7) and right (lane 11 – 15) results were used for the reproducibility confirmation.


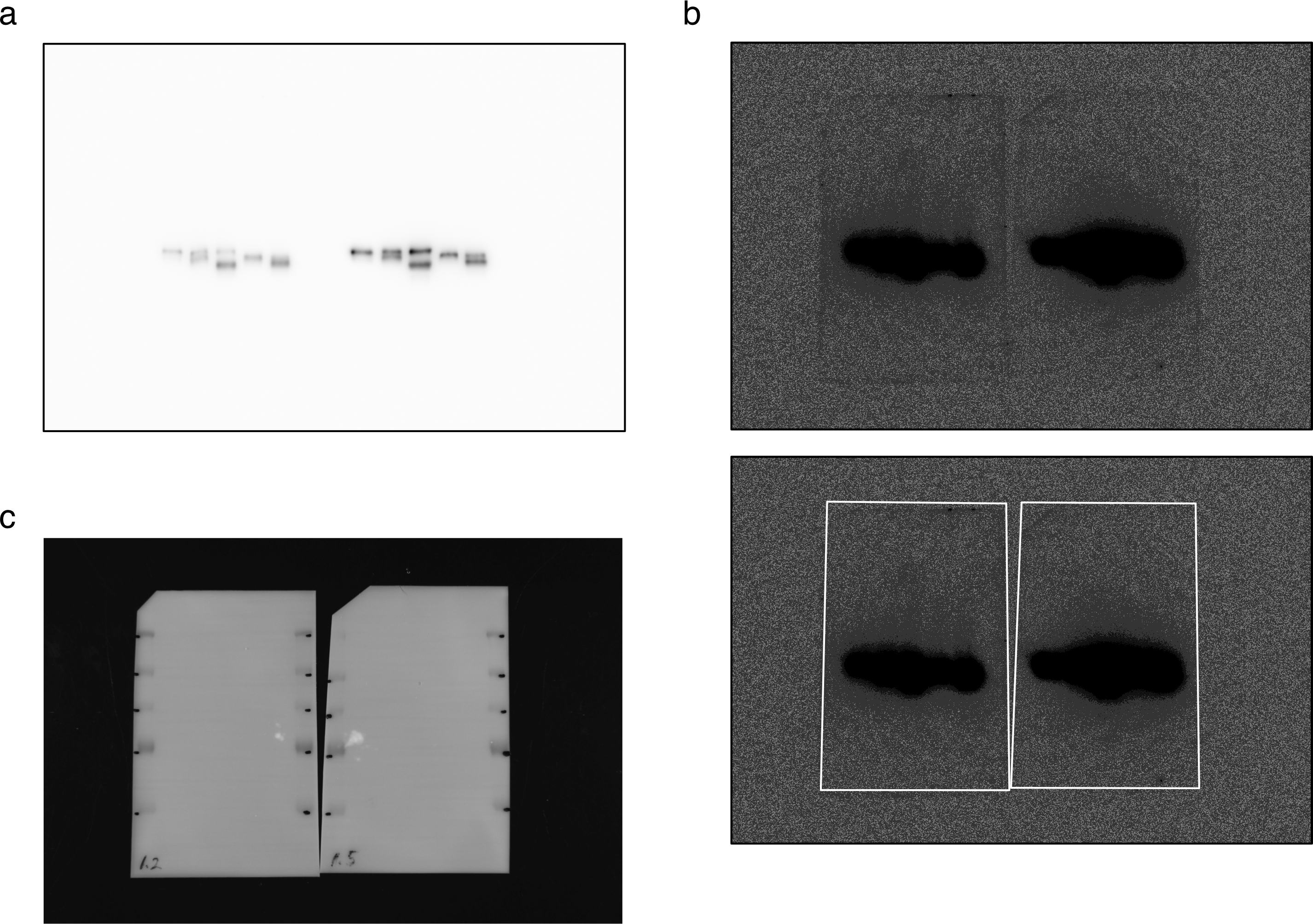


Figure S2

The full-length blot of anti- HRG western blotting analysis using plasma collected from horses with each genotype. Shown are the presentative photo of five independent experiments. (a) Unprocessed photo of western blotting analysis. (b) Contrast and brightness modified images to show the edge of membranes. The white squares in the bottom photo indicate the edge of the membranes. (c) Visible light photograph of the membranes. Left and right membranes were used for the reproducibility confirmation. The clipped photo of Figure S2a was used in Figure 4.


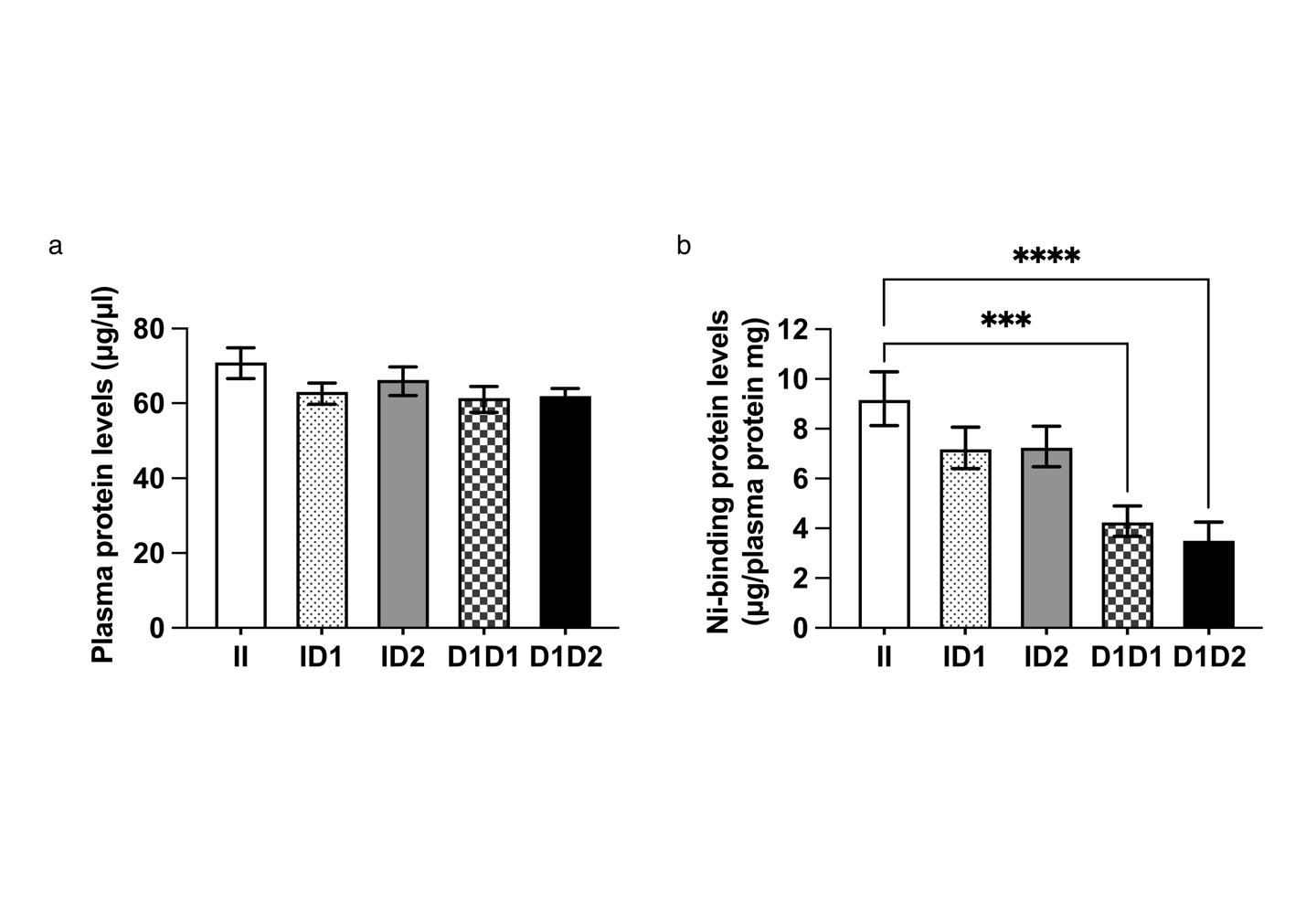


Figure S3

Plasma total protein and eHRG levels. (a) Total protein levels in the plasma isolated from horses with each genotype. (b) Amount of Ni-sepharose binding protein in the plasma of horses with each genotype. Ni-binding protein was expressed as weight per mg of total plasma protein. Each column of the graph shows the mean ± standard error. N = 10 in each group. ****p* < 0.0005, *****p* < 0.0001. II: Insertion/insertion, ID1: insertion/ 45 bp deletion, ID2: insertion/ 90 bp deletion, D1/D1: 45 bp deletion/45 bp deletion, D1D2: 45 bp deletion/ 90 bp deletion.
